# Free temporal fascia flap to cover soft tissue defects of the foot: a case report

**DOI:** 10.3205/iprs000060

**Published:** 2015-01-13

**Authors:** Martin Schreiber, Adrian Dragu

**Affiliations:** 1 Klinik für Plastische und Handchirurgie mit Schwerbrandverletztenzentrum, Klinikum St. Georg gGmbH, Leipzig, Germany

**Keywords:** temporoparietal fascia flap, flap reconstruction and foot, reconstruction foot, lye acid surgery, flap reconstruction, defect lower extremities, trauma foot

## Abstract

Severe soft tissue defects as a result of lye contamination remain a huge challenge in the interdisciplinary approach of trauma surgeons and plastic surgeons. Free tissue transfer is a suitable surgical option for successful reconstruction of form and function of defects in the distal parts of the lower extremities. We report the successful two-stage reconstruction of a full thickness lye contamination at the dorsum of the foot with a free temporoparietal fascia flap covered with a split-thickness skin graft from the thigh. The described method is a suitable operative alternative to anterolateral thigh flaps or other thin fascia flaps regarding flap harvest and donor site morbidity and should be considered in the portfolio of the plastic surgeon.

## Zusammenfassung

Laugenverätzungen am Vorfuß sind eine häufige Ursache für schwere Schädigungen der Weichteile an einer sensiblen Lokalisation dar. Aufgrund der tiefgehenden Kolliquationsnekrose erfordern diese Verletzungen oft den freien mikrochirurgischen Gewebetransfer. Im Falle einer drittgradigen Laugenverätzung des Vorfußes mit freiliegendem Sehnengewebe ist die zweizeitige Rekonstruktion durch adäquates Debridement und definitive Defektdeckung durch die freie mikrovaskulär anastomosierte Temporalisfaszienlappenplastik mit gleichzeitiger Spalthauttransplantation eine gute Alternative zur ALT-Lappenplastik oder anderen dünnen Faszienlappen. Aufgrund der sicheren Präparation sowie der geringen Hebemorbidität eignet sich die hier vorgestellte Methode sehr gut zur Defektdeckung am Fußrücken.

## Introduction

Soft tissue defects of the foot and ankle region are usually the result from mechanical, chemical or thermal trauma or are a consequence of systemic disorders with reduced perfusion, trophic dysfunction and soft tissue infections [1]. The successful reconstruction of form and function in the distal parts of the lower extremities remain challenging. In case of full-thickness burns or chemical contamination with consecutively exposed tendon material only vascularized tissue may sufficiently cover the profound defect. In the course of microsurgical progress there are several establishes options for soft tissue reconstruction, such as local perforator flaps, pedicled flaps and more recently microvascular free flaps. There are numerous studies describing soft tissue reconstruction of the dorsum of the foot with several local and pedicled flaps but to our knowledge there are only few reports of the use of a microvascular free temporoparietal fascial flap for reconstruction of full-thickness lye contamination of this precarious body region [2], [3], [4], [5], [6], [7]. This report describes the surgical treatment and short-term follow-up of a patient with full-thickness chemical burn injury of the dorsum of the foot.

## Case presentation

**Figure 1:**
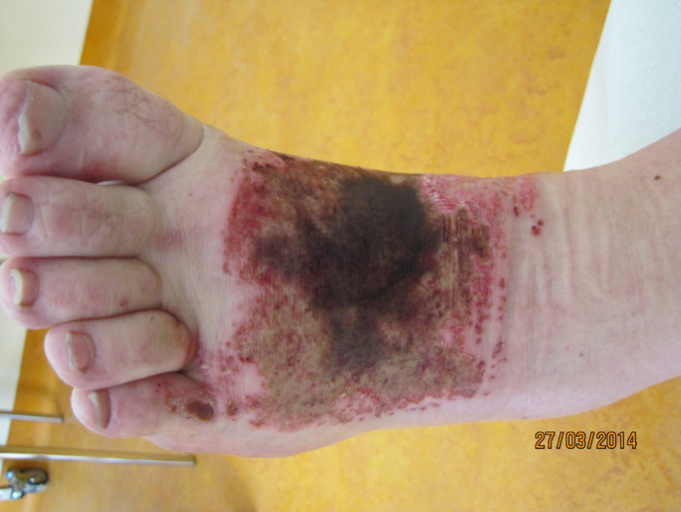
Liquification necrosis

**Figure 2:**
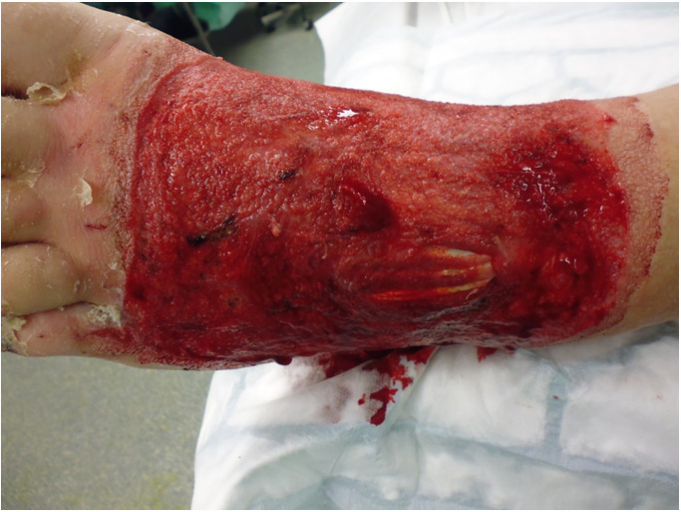
After debridement and wound bed preparation

The 24-year-old male was referred from an external hospital after a job-related chemical injury to the dorsum of his left foot. He accidentally poured an unknown amount of caustic soda solution over his foot. The emergency treatment was performed in a general hospital near to the patient’s place of residence. He was admitted to our Burns Unit the following day. Patient examination showed extensive necroses from the ankle region until the distal third of the dorsum of his left foot and the metatarsals. Planta pedis, heel and toe region remained intact (Figure 1 (Fig. 1)). Due to the extent of the clinical evidence we decided to perform a two-stage surgical approach with extensive debridement including intercurrent vacuum assisted therapy and reconstruction with a free flap. During surgical debridement we discovered a full-thickness liquefactive necrosis involving the tendons of the Musculus extensor digitorum longus (Figure 2 (Fig. 2)). Covering the defect with a skin graft was not appropriate in this situation. After preoperative arrangements and preconditioning of the defect surface with the use of continuous vacuum assisted therapy we performed microvascular free temporoparietal fascial flap transfer on our patient. 

**Figure 3:**
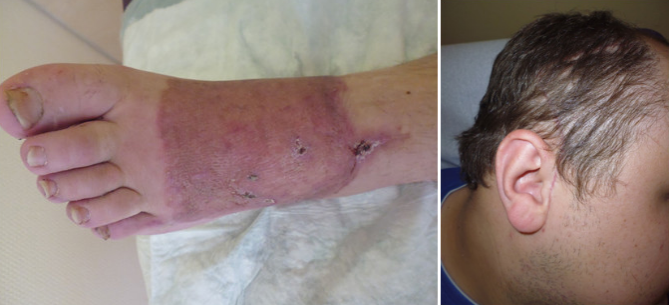
Nine weeks post-op

**Figure 4:**
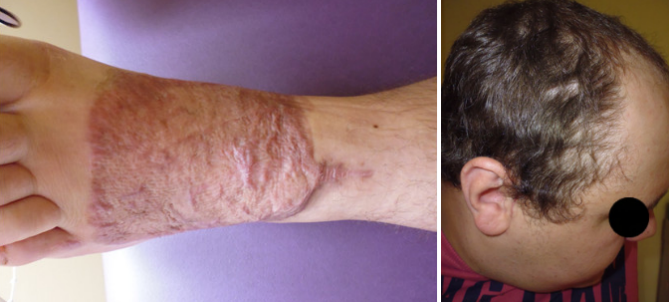
Five month post-op

**Figure 5:**
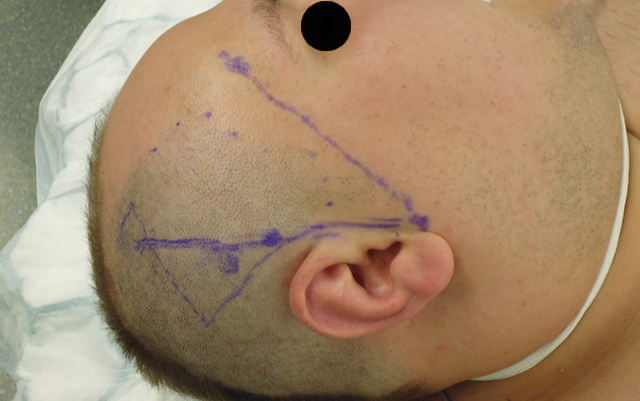
Intraoperative marking

No intra- and immediate postoperative complications occurred. The flap and the partial-thickness skin graft, which was used for flap coverage, showed no signs of inflammation. The donor sites at the right temporoparietal region and on the left thigh showed no signs of inflammation or neurologic disorders of the fascial or temporal nerve. The patient underwent early physiotherapy for remobilization. He was also assorted of compressive clothes to remodel the flap on his left foot. He was discharged to outpatient care on the 28^th^ day after admittance in a healthy condition with good scarring. The initial care was performed by our outpatient unit with weekly follow-up visits and short-term visits after 9 weeks (Figure 3 (Fig. 3)) and 5 months post surgery (Figure 4 (Fig. 4)). Given full weight bearing the patient did not complain of any disturbance. There were no signs of hypertrophic scarring at the donor site with minimal hyperaemia. We did not discover any signs of paraesthesia or functional disorder in the facial region, also the scar in the temporal region remained unremarkable.

### Surgery

**Figure 6:**
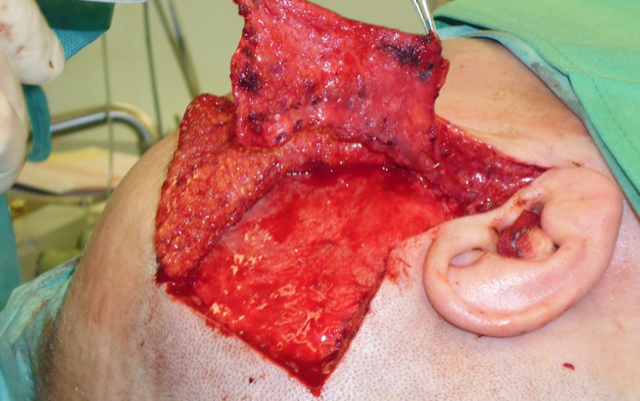
Temporoparietal fascial flap

**Figure 7:**
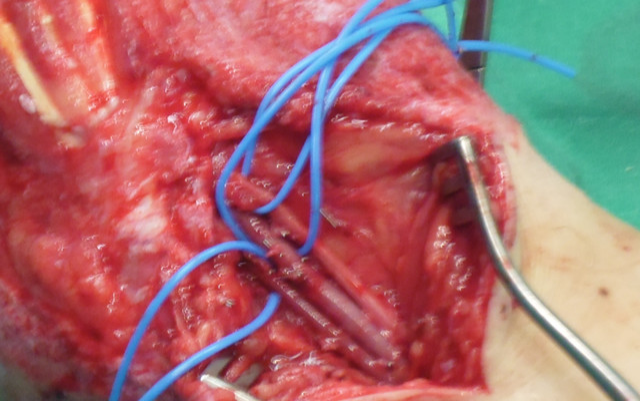
Recipient site

**Figure 8:**
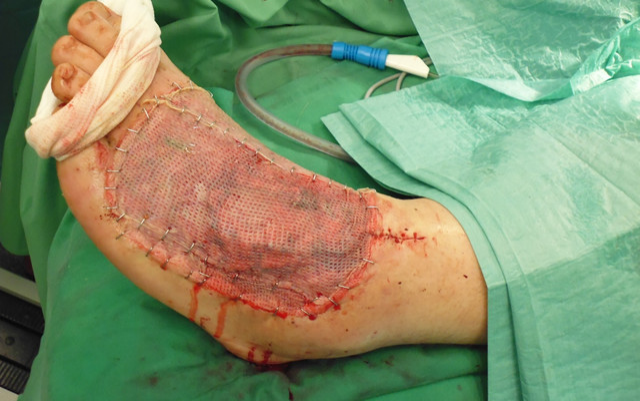
Temporoparietal fascial flap with skin-graft

Surgery was performed as a two-team-approach. We decided to perform an end-to-side anastomosis to the dorsalis pedis artery after preoperative identification of three sufficient vessels in the patient’s left lower extremity with Doppler ultrasound. Pre- and intraoperative Doppler ultrasound measurement of the temporal region identified the superficial temporal artery with its bifurcation to the anterior and posterior part. We chose a preauricular incision and dissected the temporal fascia under constant surveillance of the superficial temporal artery and without crossing Pitanguy’s line (Figure 5 (Fig. 5) and Figure 6 (Fig. 6)). Simultanously the preparation of the recipient vessels was performed, including the dorsalis pedis artery with two concomitant veins (Figure 7 (Fig. 7)). The flap measured 10 by 6 centimetres, after the flap harvest was successfully performed we used a drainage and a resorbable intradermal running suture to close the defect. Under microscope the end-to-side anastomosis was performed before suture of the temporoparietal fascial flap. Eventually we took a split-thickness skin graft from the patient’s ipsilateral thigh and covered the flap (Figure 8 (Fig. 8)). 

## Discussion

Profound soft tissue defects of the dorsum of the foot remain a huge challenge for plastic surgery. The reconstructive portfolio includes e.g. local perforator flaps, perforator-propeller-flaps, pedicled flaps or microvascular free tissue transfer. The aims of reconstructive surgery for this kind of defects are adequate tissue coverage with vascularized tissue, the reconstruction of the aesthetic and functional unit of the foot with a proper quality of life afterwards. Hence, very thin fasciocutaneous flaps are preferred [2]. Pedicled flaps for tissue coverage of the dorsum of the foot may be a feasible option, but they are usually inferior to the free flap not only functionally but also from an aesthetic point of view. Dhamangaonkar et al. examined 109 cases for defect reconstruction of distal tibial defects and defects of the dorsum of the feet. All patients received surgery with a pedicled sural flap. The authors described a failure rate of 11% with good advantages in terms of operating time and technical feasibility. In contrast they admit the obvious aesthetic deficit in donor and recipient site. In addition sural flaps remain functionally challenging with regards to orthopaedic shoe supply [3]. 

Recently Hallock published very interesting data about a perforator-propeller-flap based on a distally pedicled metatarsal artery flap which may be useful for reconstruction of very distal toe defects and defects of the dorsum of the foot [4]. The wound closure of donor site region with increased tension may be challenging as well as the need for an adequate perforator. In our opinion full-thickness lye liquification necrosis may be unsuitable for this kind of defect reconstruction with regards to vessel quality, poor success rate and an elevated risk of infections. 

A well established method for reconstruction of defects of the distal tibia and feet of slender patients is the microvascular free anterolateral thigh flap (ALT) based on perforators of the descending branch of the lateral femoral circumflex artery or the free serratus fascia flap [5], [6], [7]. A good functional outcome with decent donor site morbidity may be reached through technically safe anastomoses either end-to-end or end-to-side dependent on vascular supply. Admittedly this surgical option may at stake due to heterogeneous and increasingly overweight patients in parts of Middle and Western Europe, which emphasizes the need for alternatives. Rausky et al. described a chimeric thoracodorsal perforator flap for foot reconstruction based on a thin fasciocutanous thoracodorsal artery perforator flap combined with an anterior serrate flap [8]. They could achieve a good functional outcome with proper weight bearing of the heel in a circular defect zone of the foot. 

Due to the extensive defect of our young but overweight patient we did not chose local flap options. Patient factors such as weight and postoperative weight bearing requirements as a very young patient led us to perform surgery with the microvascular free temporoparietal fascial flap with end-to-side anastomosis to the tibal dorsal artery. With this option we achieved an optimal consensus of form and function of the foot on the one hand and the aesthetic outcome on the other hand. The short-term follow-up of our patient shows a good and plane scarring, optimal donor site outcome and a very good postoperative function. Our results are in conformity with the experiences of Duteille et al., who showed similar results on twelve consecutive patients [9]. All patients received free temporoparietal fascial flap surgery for reconstruction of profound defects of the dorsum of the foot. No vascular complications and no flap failure were detected within the study with an overall complication rate of 25%. One patient suffered from burn injury on the skin-graft area due to a lamp, one patient received a revision of split-thickness skin grafting and another patient developed selective alopecia in the donor site region. We did not observe any of those complications in our patient. Patients are thoroughly informed about the possibility of selective alopecia. If this occurs to be a concern, transplantation of follicular units and z-plasties may be offered. 

The complexity of soft tissue reconstruction of the foot arises with a relative high incidence of flap failure and secondary major amputations of the foot or distal lower leg in a lot of patients. One reason leading to amputation may be limited capacity for plastic reconstructive surgery and therefore limited surgical expertise in smaller health centers. A grave reason may also be a high complication rate of chronically ill patients with a lot of comorbidities leading care-givers to more definite options such as amputations. Ligh et al. from Duke University recently compared a group of patients which received free flaps to a group of patients which received local flaps [10]. According to their findings both patient groups showed a relative high rate for major complications resulting in amputation. 

Consistent with the findings of Duteille et al. we deem the microvascular free temporoparietal fascial flap for a good operative option in patients with profound defects of the dorsum of the foot. It may expand the portfolio of the plastic surgeon in reconstructive surgery in this body region. 

## Notes

### Competing interests

The authors declare that they have no competing interests.
